# Guy’s Hospital Reports

**Published:** 1888-03

**Authors:** 


### GUY'S HOSPITAL REPORTS.

Volume XLIV, 1887—edited by N. Davies-Colley and W. H. White—contains, as usual, many valuable papers by different writers. Of some of which *abstracts* have been prepared for the *Journal and Examiner*. They are of sufficient general interest to the profession to merit special mention.

W. Arbuthnot Lane, M. S., in a long article on "The Causation, Pathology, and Physiology of several of the Deformities which Develop during Young Life," attempts to consider more particularly the causation and pathology of the deformities, because he believes the accepted teaching largely incorrect in regard to these points. He says that "*the surgical pathologist has but too frequently mistaken the cause for the effect*," and "*therefore it is of much importance, as regards treatment, that we should obtain as thorough and as complete a knowledge as possible of the factors which determine the development and progress of deformities, and of the influence exerted by each*."

Among the supposed factors in the causation of knock-knee which the author considers incorrect, are—

1. Yielding of the internal lateral ligament.

2. Union at an early date of the outer portion of the lower epiphysis with the femur.

3. Contraction of the biceps tendon.

These the author thinks are effects, not causes, and that the real cause of the deformity is "the habitual or very frequent assumption of the position of rest of that joint."

The discussion of flat-foot occupies twenty-six pages. The author considers rotation of the os calcis inwards the chief determining factor in this deformity. The first factor which is solely and primarily responsible for the condition is a general want of tone and vigor. The discussion of this deformity is extremely technical, and the suggestions for treatment are along the line of the anatomical conditions present, and so full of detail that a condensation cannot do them justice.

Twenty-five pages are given to deformities of the spine. Attention is called to the fact that a direct backward displacement of the shoulders will effect but little change in the deformed column in cases of excurvation, and yet, this displacement, and one also slightly downward, are the only ones exerted by the braces usually constructed for these cases. The effect desired is to displace the weight of the head and shoulders as far backwards as possible. "This can be done by simply approximating the shoulders by means of powerful elastic bands attached to shields encircling them."

"In the case of the young female in whom we suspect the presence of sacral deformity we may develop a tendency to its removal by powerfully over-extending the hip and sacro-iliac joints by placing a large sandbag beneath the pelvis when the patient is occupying the supine position." In a case of typical dorsal excurvation developed during growing life the author discovered the following described anatomical peculiarity in the dissecting room, " developed in the lower part of the ligamentum nuchæ, and intimately connected by ligament to the prominent spinous process of the seventh cervical vertebra, a conical piece of bone about an inch and a quarter long. This peculiar pathological condition lends additional weight to the claim that in these cases there is a special strain upon, and consequent marked hypertrophy of, the ligamentum nuchæ."

The opening remarks in what he has to say about lateral curvature of the spine, represent so clearly the author's theory of the formation of all of these deformities acquired during young life that they may well be given here in full.

"As we analyze the changes that take place in this deformity, I think we shall arrive at the same conclusion as we did when studying the several previous deformities, namely, that there exists a natural tendency to the formation of each, since it is but the *fixation and subsequent exaggeration of a movement which is a normal one when the subject occupies the position of rest.*

" A feeble subject, such as I have already described, assumes habitually such attitudes of rest, and it is the very frequent and prolonged retention by such a person of any portion of the skeleton in its normal position of rest (that is, one which demands the smallest possible expenditure of muscular energy) that determines the permanent retention of what is normally a physiological transitory movement, and later, its progressive exaggeration. I believe that I shall be able to show that the temporary assumption of the double lateral curve by the spinal column is a normal physiological attitude, and a position of rest; that, like all positions of rest, it is most marked in the feeble subject, and that it only becomes a deformity when it remains as a permanent curve, and ceases to be recoverable in exactly the same way that a flexion of the dorsal spine is also an attitude of rest, and only becomes a deformity when it does not disappear after rest but remains permanent as a dorsal excurvation. I would point out here that the position of simple flexion of the dorsal spine, with the associated changes in the curvation of the rest of the column, is not usually assumed as a posture of rest till a period of growing life later than that in which lateral curvature develops. I will divide my case up into two headings. I wish to show—

" Firstly, that the double lateral curve is a position of rest, and consequently one which is assumed habitually by feeble children for long periods of time; and

" Secondly, that the so-called rotation of the vertebræ around a vertical axis is part and parcel of this normal and physiological process of lateral flexion; that there is nothing mysterious or obscure in the mechanism of its causation; that it is not a change which develops subsequently to that of lateral flexion, and that it is not due to any change in the form of the articular processes, or to any abnormal development of the component parts of the vertebræ on either side, but that the alteration in form which the vertebræ undergo is secondary to, and consequent upon, their movements upon one another."

The article also includes discussions of Acquired Deformities of the Toes, and Rickety Deformities. The entire article is worthy of the careful attention of all interested in orthopœdics. It is markedly original in many ways, and evidently based both upon large practical clinical experience, and unusually ample observation in the dissecting-room of the classes of acquired deformities discussed.

G. Newton Pitt, M. D., reports a case of Friedreich's Disease, of which we give here a very much condensed résumé. *Family history:* Father died of Bright's disease after a three month's illness. Mother was affected with chorea at ten years of age, but completely recovered. The patient is one of seven children. Three brothers are affected as the patient is, one less, and two more so. One sister is also worse than the patient.

In all of the cases in this family, the ataxic symptoms began at the age of fourteen to fifteen years, being worse in the legs than in the arms, and steadily increased. One of the brothers has two healthy children, the eldest of whom is ten years of age. In none of these cases have shooting pains, double vision, numbness, nor pricking sensations been present. Two brothers and his sister have suffered with weakness of the spine, and two brothers have some curvature. They are all rather pale.

The patient was first admitted at *Guy's*, May 14, 1887, and was then twenty-one years old. General health good. Seven years ago he became unsteady in his gait, and in consequence of uncontrollable movements and twitchings of his limbs, became unable to take part in games. No evidence of sudden fright or intestinal disturbance. *Present condition :* A well-nourished man, five feet ten inches high, red hair, weight 131 pounds. Tongue protruded naturally, but retracted with a jerk. Heart, lungs, liver, and spleen normal. Choreic movements are most marked when patient is in bed, and consist of a sudden jerking up of the knees, side to side movements of the feet, with pronation and supination of the forearms. When sitting up there is an occasional twist of the trunk, and, at times, twitching movements of the mouth and of the head occur. He has difficulty in rising from or sitting down upon a chair; sways to and fro when standing up, and his gait is somewhat that of a drunken man. When his eyes are closed, he is unable to touch the tip of his tongue readily. All of these symptoms are more marked when he is watched. There is no loss of muscular power. The movements are not unilateral, and cease during sleep. Has no shooting or girdle pains, no numbness in the feet. He can not stand with eyes closed and toes together. Touch and temperature sense normal. Patient remained in the hospital until December 13, 1877, and took cod-liver oil, arsenic, and at last phosphorus. Was re-admitted February 22, 1878. Up to January 15, was able to act as cashier, walking to and from work. In January, took cold, had severe cough with free expectoration. About this time, the ataxic symptoms became so bad that he could not feed himself, and he could not stand alone, and he had "flashing" pains through knees, ankles, and feet. These pains are not now present; he has lost flesh rapidly, and eyesight is dim, but he can read near and distant print well. Speech is jerky, articulation indistinct. Tongue, when protruded, is unsteady. On trying to grasp anything, the movements are unsteady, but after three attempts, he wrote his name fairly well. Has full strength in legs, but can not stand alone. In walking, the heels strike the floor first. Plantar reflexes present, sensation normal in feet and legs. Patient went home March 18, 1878, able to walk when fairly started. Re-admitted July 13, 1881. Since he has been out, has not been able to work or walk about much. A fortnight ago, he had an attack of diarrhœa, and his ataxic symptoms became much worse. *Condition on admission:* Even with his eyes open, he is unable to stand alone. When supported, he walks a few steps, shooting his legs out sideways, and bringing them down forcibly. With eyes shut, he can not touch his nose. No wasting of limbs, or loss of power, no knee-top or ankle clonus. Tongue protruded straight and steadily. Voice normal, but speech resembles that of first stage of intoxication. Sensation perfect, no numbness. All abnormal symptoms worse when he is out of sorts or tired, special organs normal.

The patient was discharged and re-admitted several times, the ataxic symptoms described gradually increasing, and he finally died at home, rather suddenly, March 21, 1885. Excepting the spinal cord the only changes of interest found at the section were a widely distributed obliterative endarteritis of the smaller arteries, with extensive chronic endo-myocarditis. A very elaborate description is given of the cord, accompanied with a lithographic plate, and followed by a condensed summary which we give in full.

1. The spinal cord is extremely small.

2. Extreme sclerosis, amounting almost to destruction of the columns of Goll, traceable from the lumbar enlargement to the floor of the fourth ventricle, where it terminates.

3. Severe sclerosis of the posterior part of Burdach's columns, in which, however, single healthy fibres are scattered; these are more numerous in the upper than in the lower part of the cord, where they are very exceptional. Slight degeneration is visible in the fasciculus cuneatus and in a bundle of fibres of the funiculus rotundus.

4. A narrow tip in Burdach's columns bounding the posterior horn and root, more especially along the anterior half, has escaped degeneration. This strip is more defined in the upper than in the lower part of the cord.

5. A diffuse sclerosis (and much less extensive than in the posterior columns) of the crossed pyramidal tracts, not varying much in intensity, but probably most marked in the dorsal region. This is not traceable higher than the commencement of the crossing of the pyramidal tracts.

6. A diffuse sclerosis of the ascending cerebellar tracts as high as the decussation of the pyramids, and a very slight and irregular sclerosis affecting scattered fibres anterior to these tracts, chiefly along the periphery and in a few sections extending along the anterior fissure.

7. Degeneration of Clarke's columns in some sections.

8. Degeneration of some of the fibres of the posterior nerve-roots, and in some sections of the posterior horns.

9. Friability and shrinkage of the affected areas.

The most noticeable feature in the naked-eye appearance of the cord was its great diminution in size in transverse section, which was as well marked in the cervical and lumbar enlargements as in the dorsal region, the posterior portions being in proportion smaller than the anterior.

There were well-marked symmetrical areas of sclerosis affecting the posterior columns, and, to a less extent, the posterior part of the lateral columns, varying in amount in different parts of the cord. The cord, however, seemed to be much smaller than could be accounted for merely by the sclerosis, and the possibility of the cord being a very small and imperfectly-developed one may be the explanation of its early degeneration.

The parts of the report which are partly reproduced here are both preceded and followed by very interesting comments upon the case reported and also of the cases of this disease which have been reported by other observers.

### OBSERVATIONS ON PROGNOSIS.

P. H. Pye-Smith, M. D., in "Observations on Prognosis," says: Of the several departments which make up the art of medicine—observation and detection, foresight, prevention, and remedy—one of the most ancient is the art of prognosis, the forecasting of future events. In the aphorisms of Hippocrates there is more of prognosis than of treatment, and prognosis has a larger share of attention than in any modern work on medicine.

Prognosis, foreknowledge of the future course and event of a disease, has for its objects either to obviate and prevent, or at worst, mitigate, an untoward event, or, on the other hand, to predict a favorable result, and thus to spare a patient a needless attempt at cure (or, as we now more modestly call it, treatment) of his malady, and to furnish him with the most pleasant and not least effectual of remedies—good hope.

Prognosis is based upon diagnosis or thorough knowledge of the disease or injury in question—of its nature and origin, of the anatomical structures which it changes, of the physiological functions it disturbs, and of the chemistry it perverts. It proceeds upon wide and accurate observation of the course of similar diseases or injuries. It is completed by critical comparison of the natural progress and event of the morbid process with two modifying factors—the state of the individual and the effect of treatment.

We now know the natural course of almost every malady. We know, for example, that stone in the bladder will sooner or later kill; that a strangulated hernia will do so more quickly, though not more certainly; that cataract will not disappear by a *vis medicatrix naturæ* any more than will an ovarian tumor; for before modern treatment made these diseases curable, authentic histories of their results were recorded. We know the average tendency of cholera and enteric fever, because these maladies have been often ill-treated, often un-treated, and are still variously treated. And, thanks to the Anti-Vaccination League, the Contagion Defence Association, the Anti-Mercurialists, and the Teetotalers, we know the natural course and the natural fatality of small-pox, of syphilis, of fevers, and of pneumonia. Thus we can estimate the value of a given plan of treatment by comparing its results, not only with those of other treatment, but also with those of no treatment at all. Happily we are able in many cases to change the natural prognosis entirely, or partially, for the better, if we can insure the intervention of rational therapeutics. With this we are not here concerned. Our inquiry relates to the prognosis of diseases apart from treatment, and especially to the modifications which the general forecast should undergo by consideration of the age, previous illness, and what used to be vaguely called the "constitution" and the "diathesis" of the individual patient.

By the "constitution" or *habitus* of the patient, I mean nothing but what can be stated in exact terms—his birth-place, parentage and race, his height and weight, girth and form of chest, the quantity and quality of his urine, the state of his heart and pulse, of his lungs and bowels, his habits of eating and drinking, his clothing, and his dwelling.

By "diathesis," disposition, proclivity, or tendency—I mean the tendency of intemperance in liquor to produce delirium tremens and cirrhosis, the disposition of rheumatic fever and of erysipelas to return, and of enteric fever to relapse, the proclivity of the children of gouty or phthisical parents to become gouty or consumptive themselves.

Of the two elements of prognosis—knowledge of the habitus, tendency, or natural course of the disease, and knowledge of the circumstances, history, and physiological state of the patient—the former is the fundamental; sure prognosis depends on accurate diagnosis. And it is possible in many instances to form a good forecast without either sight or hearing of the patient. The malignancy of hydrophobia, of carcinoma, of confluent small-pox, the fatal effects of perforation of the bowel, of extensive burns, and of intracranial tumors, admit of no hope, however doubtful, from individual circumstances; even if in some exceptional case, or from some heroic remedy, recovery ensues, it is independent of them.

But this is not true of most diseases and injuries. For example, the prognosis of diabetes, of pneumonia, typhus, compound fractures, lithotomy, is greatly determined by the individual case; so that we cannot gauge the risk of typhus, or diabetes, or lithotomy till we know the patient's age, nor judge of the effects of any operation till we have tested his urine, nor forecast the issue of an acute inflammation till we learn whether he is temperate in the use of liquor. So important is the personal element in prognosis that we may sometimes make more than a shrewd guess as to the event of a disease by observing the conditions of the patient, even when we are unable to make a satisfactory diagnosis. Hippocratic prognosis was almost entirely of this kind. In the absence of systemic post-mortem examinations, diagnosis, as we now use the term, was impossible. Yet there were certain signs which were recognized as favorable or the reverse. Subsultus tendinum, singultus, convulsions during a fever, were early recognized as being of ill omen. Sleep, returning appetite, a free discharge of urine, were observed to be usually precursors of recovery. Modern science has added little to this kind of prognosis, the power of prediction of an immediate event. Again and again we may see patients suffering from mortal diseases, from internal cancer, advanced phthisis, or cardiac lesions, rally after lying at the point of death, and again and again deceive our expectations. Cases which, it seemed, must end within an hour have lasted for days and weeks, while again and again death comes on suddenly in the midst of apparent improvement, and cases which seemed likely to last for weeks have been cut short in an hour.

The wisest and most experienced are most unwilling to hazard predictions as to time. On this branch of prognosis I have nothing to add. We are dealing in this paper, not with the immediate prospect or the probable duration, but with the ultimate result of diseases.

PROGNOSTIC APHORISMS.

Epidemic diseases are most fatal when first introduced.

Acute diseases following upon chronic are the most dangerous.

A degree of pyrexia, which is of slight importance in a child, is grave in an adult, and imminently perilous in an old man.

Typhus fever is most dangerous to persons who have passed their sixtieth or fiftieth year, less so to infants and those between thirty and fifty-five, and least dangerous to children above five and to young adults.

Small-pox in these particulars closely resembles typhus.

Whooping-cough is dangerous during infancy, and benign after five years of age.

Scarlet fever seldom takes on a malignant form when it attacks adults.

Acute lobar pneumonia has usually a favorable issue in youth and is usually fatal in advanced years.

In young adults pneumonia is rarely fatal unless the patient has disease of the kidneys or of the heart, or is of intemperate habits.

Pneumonia is also a dangerons complication of fevers or acute rheumatism.

Acute lobar pneumonia, when not fatal, leaves the lung uninjuied after recovery and the patient in good health. It is seldom or never followed by phthisis, even when it attacks the apex.

Primary acute pleurisy is not fatal, unless it is accompanied by pericarditis.

Pleurisy, if under treatment it ends in death, is secondary to tubercle or to cancer or to disease of the kidneys.

Œdema of the larynx is very seldom dangerous, œdema of the lungs is usually so.

Acute bronchitis is a frequent cause of death in young children and in old people.

Fatal bronchitis, in persons between ten and sixty years of age, is either capillary or secondary to tubercle.

Phthisis is most pernicious when it is hereditary.

Consumptive patients who lose flesh and color and appetite, with but little signs of disease in the lungs, are in a worse case than those who have marked local symptoms, but whose appetite and nutrition are good.

Hæmoptysis, even when copious, is not always of ill omen.

It is rare for hæmorrhage from either the lungs or the stomach to be immediately fatal, except it proceed from an aneurysm.

Chronic valvular disease of the heart, when it complicates phthisis, does not aggravate the latter, rather it checks its progress.

Sudden death is more frequent from aortic than from mitral lesions, in regurgitant than in obstructive disease of the aortic valves, and in stenosis than in dilatation of the mitral orifice.

Apoplexy, when ingravescent, is commonly fatal.

In apoplectic attacks, the ultimate prognosis depends chiefly upon the degree and continuance of unconsciousness, the immediate prognosis upon the degree in which respiration is affected.

Chronic diseases of the spinal cord are more likely to end favorably in women than in men.

Chorea is only fatal when the patient cannot sleep.

Malignant tumors are more rapidly fatal in the young than in the old. Cancers in the aged are sometimes exceedingly slow in their progress, and may even in rare cases atrophy.

Stone in the kidney may frequently be cured without operation. The opposite is true of stone in the bladder.

Diabetes is rapidly fatal when it occurs in young men, more curable in middle life, and of little danger in later years.

Diarrhœa is dangerous only in infants and in persons above 60 years of age.

### CASES OF HÆMORRHAGE.

In cases of hæmorrhage occurring during treatment by salicylate of soda, Lauriston E. Shaw, M. D., says: The discussion on the treatment of acute rheumatism by salicylic acid and its salts, which was opened by the late Dr. Hilton Fagge at the Medical Society in 1881, gave rise to an almost unanimous expression of opinion in its favor; nor has the mass of experience which has since been accumulating by the extensive use of these drugs in any way tended to lessen their popularity.

The theories as to the manner in which the salicylic compounds attack the rheumatic poison are likely to be varied and uncertain whilst the nature of the poison remains unknown, and with the extended experience of the last five years there is still room for doubt as to the actual effect of this drug upon the heart complications of acute rheumatism.

The difficulty in determining this latter point depends upon the variations in the frequency of cardiac affections in the different batches of cases under precisely similar conditions as regards treatment, the variation in such cases being probably partly due to the climatic, social, and hygienic surroundings of the patient. Moreover, the difficulty is added to by the impossibility of properly comparing cases under physicians whose interpretation of slight alteration of cardiac sounds may be so different. Most of us are therefore content to accept the *a priori* conclusion that, as the salicylate lessens the fever and shortens the duration of the disease, it probably diminishes the chance of endocarditis and pericarditis arising.

The toxic effects of these remedies have always been regarded as inconvenient, and by some as an actual bar to their employment. Their most strenuous opponent, Dr. Greenhow, objected to them on account of the serious cardiac depression which he had so often observed. His objections were read before the Clinical Society in 1880, but were treated lightly by most of the physicians who took part in the discussion before alluded to. Among the common toxic symptoms observed have been headache, deafness, tinnitus, vomiting, epistaxis, rashes, and various defects of the heart's action, such as slowness, intermittence, and weakness; and among the rarer, albuminuria, hæmaturia, and temporary amblyopia without retinal change.

In 1881, and since, there have occurred at Guy's Hospital three cases in which hæmorrhage took place (in one case into the retina and in the other two into the urinary tract) during the administration of salicylate of soda, and probably as a direct result of the drug.

He says: By the kindness of Dr.

Pavy, under whose care the cases were treated, I am permitted to put them on record. He then gives the histories of three cases, of which only a summary of each one is here given.

CASE 1. Acute rheumatism; mitral regurgitation; salicylate delirium; epistaxis; retinal hæmorrhage; blindness.

CASE 2. Acute rheumatism; third attack; salicylate treatment; severe delirium; hæmaturia; ecchymosis of pelvis of kidneys and bladder; death.

CASE 3. Enteric fever; administration of salicylate of soda; delirium; hæmaturia; ecchymosis of pelvis of kidneys and bladder; death.

That hæmorrhage in the way of epistaxis is a frequent result of salicylate poisoning there is abundant evidence to prove. In nearly all the abstracts of cases of acute rheumatism treated in this way that have been brought before the societies, this symptom is mentioned as occurring with more or less frequency. How this result is produced is not quite evident, although it is worthy of remark that it occurred in seven of Dr. Greenhow's fifty cases, or in 14 per cent., whereas of the 174 cases at Guy's to which I am about to refer it only occurred in eleven, or about 6.3 per cent. When it is borne in mind that in the former cases "more or less weakening of the pulse, requiring the free administration of stimulants, occurred in nearly every case," and that in the latter this symptom was comparatively rare and was hardly ever considered grave enough to necessitate alcohol, it would appear that the epistaxis, and therefore the other hæmorrhages are directly connected with the depressing effect of the drug on the circulation. Granting that the salicylate compounds given in large enough doses will produce hæmorrhage, it still remains to be considered whether in the above cases the drug was responsible for the accident.

In Case 1, in which the hæmorrhage was into the retina, although I have been unable to find a similar case recorded, there seems little reason to doubt the casual relation of the drug to the hæmorrhage. The furious delirium and bounding pulse, interrupted and temporarily relieved by a severe epistaxis, which recurred, finally passed off and left the boy partially blind, with a well-marked blood-clot covering a portion of his retina, left no doubt in the minds of those who watched the case that the drug was the cause of the accident.

In the second and third cases, in which the hæmorrhage was into the pelvis of the kidney and the bladder, the similarity of the lesions is very striking, and in making the post-mortem on the second case I was at once reminded of the former, which I had seen Dr. Goodhart examine four years before.

Of the few reports of cases dying certainly or doubtfully from the results of salicylate poisoning that I have been able to consult, no mention of such lesion is made. On the other hand, transient hæmaturia has been recorded. Mr. Mllican, at the Medical Society, in 1882, mentioned a case in which purpura and hæmaturia seemed to have resulted from medicinal doses of salicylate of soda. But in this case the patient had previously suffered from "hæmoptysis as vicarious menstruation," which throws some slight doubt on its true importance.

Among the 174 cases before mentioned, besides Case 2, which is included among them, there is another case in which hæmaturia was present for some time, and in which there also occurred epistaxis.

In Case 2 there can be no doubt that the patient suffered severely from the toxic effects of the drug, as shown by the deafness, headache, "peculiar noises" in the ears, and delirium. It is not possible to accurately estimate the quantity of medicine she took, as it was given rather irregularly and now and then stopped on account of the delirium, but it is probable that she took not less than 240 grains of the soda salt and 400 grains of salicine during the four days she was under treatment. The hæmaturia appeared when she had taken at least three-quarters of the whole amount and at a time when the other poisoning symptoms were most marked. Whether the rheumatic or the salicylic poisons were the cause of death—and there are grounds for the former hypothesis in the severity of the attack and the fact that rheumatism does, though rarely, kill without hyperpyrexia, and without visible pathological lesion—there can be little doubt that it was the latter which caused the lesions in the kidney and bladder.

Case 3, taken by itself, affords much weaker evidence of the relation of the drug to the symptom, but taken in connection with the second it both gives and receives additional weight. Its weakness is two-fold—firstly, in that the symptoms of poisoning, headache, vomiting, and delirium, which are mentioned in the report, were already present on admission as a result of the typhoid from which she was suffering; secondly, in that hæmaturia is by no means uncommon in typhoid fever.

With reference to the first point it should be noticed that the salicylate was early changed to a salicine, a pretty certain indication that the physician or house physician who ordered the change considered that the patient was suffering from salicylism. The patient, moreover, received fairly large quantities of the drugs, *i. e.*, at least 60 grains of the soda salt and 480 grains of salicine in five consecutive days.

With reference to the second point, in nearly all cases in which typhoid proves fatal when complicated with hæmaturia it is found that the hæmorrhage is due to a general nephritis, and not to the condition of ecchymosis which bears so marked a resemblance to Case 2. There is little doubt that the patient succumbed to the typhoid fever, and as to the extravasations of blood one cannot perhaps say more than that they probably resulted from salicine.

Those physicians who are inclined to look upon the toxic effects of salicine as mere inconveniences, which are not to be considered beside the great advantages obtained in combating the rheumatic poison, would not look with indifference upon such accidents as are above described. But they are very rare and should no more deprive us of the use of this powerful weapon than do the accidents that happen in the administration of anæsthetics. They should perhaps suggest caution in pushing the drug beyond its physiological extent, of which we get early warning in the deafness and headache which occur.

It has been suggested that the poisonous symptoms are due to some bye-products in the synthetic preparation of salicylate of soda, and that they therefore would not occur during the administration of natural salicine or salicylic acid. But as the soda salt is more active in producing toxic symptoms, so is it more powerful in relieving the rheumatic pains and reducing the temperature, and at Guy's, at least, the soda salt is almost exclusively used, except in cases in which there seems to be some undue sensitiveness to the drug, when salicine is substituted.

It does not, therefore, seem likely at present that the first place will be given to the natural drug, nor, as I shall presently show, did improved methods of manufacture of the synthetic drug, if such improvements there have been, make it less active in producing the more common symptoms in 1886 than it was in 1881.

In looking through the cases of acute rheumatism occurring at Guy's in the years 1881 to 1886, inclusive, for evidence of similar accidents to those which form the subject of this paper, I have tabulated, for the sake of comparison, the results in the way of toxic symptoms in the cases in the first and last years. Every case of acute rheumatism of which the report is sufficiently full to estimate the dose of medicine is included. In so many cases both salicylate of soda and salicine were given, and in so few was salicine only given, that no attempt has been made to separate the results of the two drugs. All cases in which no mention of any toxic symptoms is made have been considered as not presenting them, although the fact that in many such cases salicine was early substituted for the soda salt suggests that such symptoms were present but not noticed by the clerk; but the unavoidable error due to this want of observation is likely to have been as great in one year as the other.

In both years the soda salt was much the greater favorite, there being only five or six cases in each year in which salicine only was given throughout the treatment. The dose has not practically changed, the commonest dose to begin with being in both years twenty grains every three hours. The soda salt used in both years was, as it has always been at Guy's, that artificially prepared by a patent process in Germany and received at the hospital in the original jars.

In 1881 there were 102 available cases, and in 1886 seventy-two.

The following table shows the relative frequency of the common toxic effects.

| | 1886. | | 1881. |
|---|---|---|---|
| | Cases. | Per Cent. | Cases. |
| No toxic effects mentioned | 23 | 32 | 40 |
| Toxic effects | 49 | 68 | 62 |
| Delirium | 12 | 16 | 21 |
| Deafness | 28 | 38 | 33 |
| Vomiting | 17 | 23 | 15 |
| Tinnitus | 13 | 18 | 16 |
| Headache | 21 | 29 | 12 |
| Epistaxis | 5 | 6 | 6 |
| Irregular or slow pulse | 9 | 12 | 4 |
| Albuminuria | 2 | — | 4 |
| Hæmaturia | 1 | — | 1 |
| Retinal Hæmorrhage | — | — | 1 |
| Urticaria | — | — | 1 |

The frequency of occurrence of the various symptoms in the seventy-two cases in 1886, has been reduced to percentages in order to the better compare them with the 102 cases in 1881.

The most notable differences are in delirium, which is about 5 per cent. less frequent, and in vomiting, which is about 8 per cent. more frequent in the latter than in the former year. Headache and alterations in the pulse, which are both much more frequent, the one 17 and the other 8 per cent., are of less importance on account of the many possible causes other than the medicine which might be responsible for their occurrence, and also because they are symptoms which could more likely be omitted by the clerk.

The main fact which the table shows is that whatever alteration may have taken place in the drug or its method of administration, the chief toxic symptoms are as common now as when it was first introduced, and whatever danger there may be of such accidents as have been related is as great now as it ever was.

